# Channeling of Carbon Flux Towards Carotenogenesis in *Botryococcus braunii*: A Media Engineering Perspective

**DOI:** 10.3389/fmicb.2021.693106

**Published:** 2021-07-29

**Authors:** Iqra Mariam, Mukul Suresh Kareya, Mohammed Rehmanji, Asha Arumugam Nesamma, Pannaga Pavan Jutur

**Affiliations:** Omics of Algae Group and DBT-ICGEB Centre for Advanced Bioenergy Research, Industrial Biotechnology, International Centre for Genetic Engineering and Biotechnology, New Delhi, India

**Keywords:** *Botryococcus braunii*, carotenoids, isoprenoids, methyl erythritol phosphate, phylogenomics, squalene

## Abstract

Microalgae, due to their unique properties, gained attention for producing promising feedstocks having high contents of proteins, antioxidants, carotenoids, and terpenoids for applications in nutraceutical and pharmaceutical industries. Optimizing production of the high-value renewables (HVRs) in microalgae requires an in-depth understanding of their functional relationship of the genes involved in these metabolic pathways. In the present study, bioinformatic tools were employed for characterization of the protein-encoding genes of methyl erythritol phosphate (MEP) pathway involved in carotenoid and squalene biosynthesis based upon their conserved motif/domain organization. Our analysis demonstrates nearly 200 putative genes showing a conservation pattern within divergent microalgal lineages. Furthermore, phylogenomic studies confirm the close evolutionary proximity among these microalgal strains in the carotenoid and squalene biosynthetic pathways. Further analysis employing STRING predicts interactions among two rate-limiting genes, i.e., phytoene synthase (PSY) and farnesyl diphosphate farnesyl synthase (FPPS), which are specifically involved in the synthesis of carotenoids and squalene. Experimentally, to understand the carbon flux of these rate-limiting genes involved in carotenogenesis, an industrial potential strain, namely, *Botryococcus braunii*, was selected in this study for improved biomass productivity (i.e., 100 mg L^–1^ D^–1^) along with enhanced carotenoid content [0.18% dry cell weight (DCW)] when subjected to carbon supplementation. In conclusion, our approach of media engineering demonstrates that the channeling of carbon flux favors carotenogenesis rather than squalene synthesis. Henceforth, employing omics perspectives will further provide us with new insights for engineering regulatory networks for enhanced production of high-value carbon biorenewables without compromising growth.

## Introduction

The rapid increase in energy consumption globally along with greenhouse gas emissions and depletion of fossil fuels has raised the requirement for the development of sustainable renewable energy sources (Davis et al., 2011; Lim et al., 2015). This leads to the increased production of biodiesel in recent times with annual production reaching over billions of liters (Ogunkunle and Ahmed, 2019). The conventional source of biodiesel production from *Pongamia pinnata*, *Jatropha curcas*, etc., may not be sustainable due to competition of land in terms of fuel vs. food (Chisti, 2007; Ogunkunle and Ahmed, 2019). Nonetheless, microalgae in due course of time emerged as a feasible alternative for biodiesel production because of their higher yields, their efficient light channeling leading to better photosynthetic efficiencies, their rapid reproduction cycles, and their ability to grow in variety of water resources (brackish, saline, and even wastewaters) (Guedes et al., 2011; Ratha and Prasanna, 2012; Abdelaziz et al., 2013; Leite et al., 2015). Other advantages include their ability to synthesize certain high-value renewables (HVRs) such as long-chain polyunsaturated fatty acids (LC-PUFAs), carotenoids such as β-carotene, astaxanthin, lutein, and isoprenoids like squalene, which are compounds of nutraceutical and pharmaceutical relevance (Spolaore et al., 2006; Raja et al., 2008).

Microalgae tend to accumulate lipids in the form of triacylglycerols (TAGs) and starch as carbon storage compounds in nutrient deprivation and other abiotic factors such as light, temperature, and carbon supplementation in the form of CO_2_ and bicarbonate (Gardner et al., 2012; Menon et al., 2013; Abdelaziz et al., 2014; Tsai et al., 2014; Srinivasan et al., 2018). However, enhanced lipid accumulation in nutrient-deprived condition is concomitant with retarded growth leading to lower biodiesel productivity, which is a major bottleneck (Tsai et al., 2014; Srinivasan et al., 2018). Microalgae are also known for the production of various other HVRs such as eicosapentaenoic acid (Szklarczyk et al., 2019), docosahexaenoic acid (DHA), vitamin E (α-tocopherol), carotenoids, and squalene (Singh et al., 2020; Mariam et al., 2021; Paliwal and Jutur, 2021). The co-production of such HVRs will further be a cost-effective addition in terms of commercial value of biodiesel production from microalgae (Dewapriya and Kim, 2014; Jutur et al., 2015).

Despite the structural difference existing between carotenoids and squalene, both compounds share a common intermediate, i.e., geranyl geranyl diphosphate (GGPP), formed by condensation of two isoprene units: isopentenyl diphosphate (IPP) and dimethylallyl phosphate (DMAPP) (Matsushima et al., 2012; Zeng and Dehesh, 2021). This condensation reaction of IPP and DMAPP occurs within the chloroplast through the methyl erythritol phosphate (MEP) pathway (Scodelaro Bilbao et al., 2020). Moreover, the MEP pathway and lipid biosynthesis participate in a complex crosstalk between each other and appear to be upregulated upon carbon supplementation (Kareya et al., 2020; Scodelaro Bilbao et al., 2020). Overall, the MEP pathway encompasses an extensive list of compounds, owing to its comprehensiveness and because its regulatory hubs in microalgae have largely remained elusive. Henceforth, it is important to understand the mechanisms in microalgae that alter the regulation of the specific pathways upon carbon supplementation.

In this context, in the present study, we have selected an industrial potential strain, i.e., *Botryococcus braunii*, a colonial microalga belonging to the Trebouxiophyceae family, known for production of squalene and carotenoids (Uchida et al., 2018). As a result, this microalga has gained tremendous commercialization due to its high isoprenoid and lipid contents. However, its high doubling time and difficult handling present a major bottleneck for mass cultivation in open ponds (Hirano et al., 2019). Moreover, the cellular machineries are inadequately understood in this microalga, and there is a need to reveal the regulation of MEP pathway. In the present work, our aim was to predict the changes within the molecular profiles occurring when supplemented with carbon, thus filling the gaps and providing insights in understanding the intricate networks of the MEP pathway for the production of squalene and carotenoids. Carbon supplementation enhances the photosynthetic carbon fixation in microalgae generating glyceraldehyde 3-phosphate (G3P) pool through the Calvin–Benson cycle. This G3P pool along with pyruvate is the precursor molecule for MEP pathway. Furthermore, in several microalgal species such as *Microchloropsis gaditana*, *Chlorella pyrenoidosa*, and *Dunaliella salina*, carbon supplementation in the form of CO_2_ or sodium bicarbonate has been reported to enhance carotenoid production (Sampathkumar and Gothandam, 2019; Kareya et al., 2020; Xi et al., 2020). Studies on phylogenomics demonstrate the evolutionary relationship at the genetic level of these protein-encoding genes involved in carotenogenesis among divergent microalgal lineage. Additionally, quantification of the HVRs in *B. braunii* reveals the channeling of carbon flux toward squalene and carotenoid biosynthesis when supplemented with additional carbon without compromising growth. Overall, this study reveals that a new approach of media engineering; i.e., carbon supplementation enhances the photosynthetic performance in microalga *B. braunii*, which further helps us to understand the crosstalk between different metabolic pathways involved in enhanced production of biomass, biofuels, and biorenewables (B^3^).

## Materials and Methods

### *In silico* Analysis

#### Identification of Putative Genes Involved in Methyl Erythritol Phosphate Biosynthetic Pathway

**FIGURE 1:**
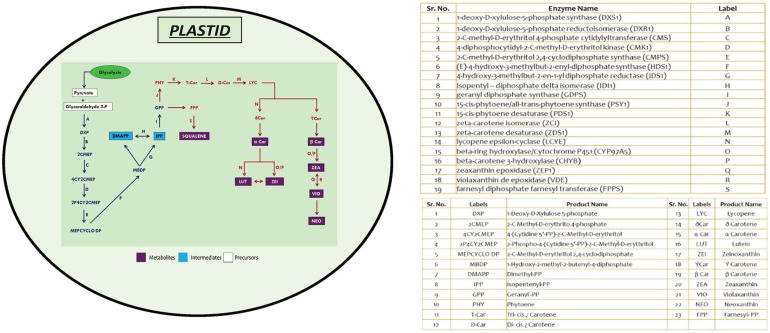
An overview of methyl erythritol phosphate (MEP) pathway in microalgal cells depicting carotenoid and squalene biosynthesis. Genes marked in blue are initial steps of MEP pathway, and those in red are involved in high-value renewables (HVR) (squalene and carotenoids) synthesis.

The protein-encoding genes involved in carotenoid biosynthesis was retrieved from the Kyoto Encyclopedia of Genes and Genomes^1^ (Kanehisa et al., 2010). The reference dataset for the above-mentioned genes was obtained from *Chlamydomonas reinhardtii*, which was subjected to BLASTp^2^ (Altschul et al., 1990) with a set threshold *e*-value of 1e^–10^. Homologs were identified in different microalgal species, as these species have well-annotated genomes available, which include *Volvox carteri*, *Coccomyxa subellipsoidea*, *Micromonas pusilla*, *Micromonas commoda*, *Ostreococcus tauri*, *Ostreococcus lucimarinus*, *Aurantiochytrium limacinum*, *B. braunii*, *D. salina*, *Nannochloropsis gaditana*, *Phaeodactylum tricornutum*, and *Chlorella variabilis*. The gene set was chosen based on query coverage (>60%), percentage identity (>30–40%), and *e*-value scores (Supplementary Table 1). A schematic representation of MEP biosynthetic pathway existing among microalgal lineages was illustrated (Figure 1; Kanehisa et al., 2010), depicting the presence of various functional genes involved in the carotenogenesis, facilitating better understanding of the individual components involved in the production of these high-value carbon molecules.

#### Prediction of Subcellular Localization/Motif and Domain Organization

*In silico* predictions for protein localization was performed using four online prediction software, namely, TargetP (Emanuelsson et al., 2000), Cello (Yu et al., 2004), ngLOC (King and Guda, 2007), and WoLF PSORT (Horton et al., 2007). Each protein sequence was examined using all four prediction algorithms, and the protein with the highest consensus predicted location was assigned to it. Motif prediction for the protein sequences was performed using the MEME suite^3^ (Bailey et al., 2015). Parameters used for the motif prediction consisted of number of sites, 2–600; number of repetitions, 0–1 per sequence; width limit, 6–50; and maximum number of motifs, up to 3. The domain was predicted using HMMER v3.3.2 (Potter et al., 2018)^4^ web server and constructed using an online ExPASy tool MyDomains-Image Creator.

#### Physico-Chemical Properties/Guanine–Cytosine Content Characterization of Protein-Encoding Genes

Computational analysis of physico-chemical parameters was performed using ExPASy’s ProtParam server that computes molecular weight, aliphatic index, instability index, grand average of hydropathy (GRAVY), and isoelectric point (pI) (Gasteiger et al., 2005). The guanine–cytosine (GC) content was determined using the GENSCAN web server (Burge and Karlin, 1997).

#### Evolutionary Phylogenomics and Subcellular Network Prediction

To comprehend the evolutionary relationships among the protein-encoding genes in the studied microalgal species, a phylogenetic tree was constructed using MEGA X software (Kumar et al., 2018). After alignment of the sequences by ClustalW, the phylogenetic tree was constructed using neighbor-joining (N-J) method, and the evolutionary distances was computed using the Jones–Taylor–Thornton (JTT) matrix-based method (Shaikh et al., 2020). The parameters were as follows: phylogeny test, bootstrap method; no. of bootstrap replications, 1,000; and gaps/missing data treatment, pairwise deletion. Interacting networks of carotenoid biosynthesis proteins were constructed using the STRING database^5^ (version 11.0; Szklarczyk et al., 2019).

### Functional Analysis of Isopentenyl Diphosphate Pathway in Microalgal Species

#### Culture Conditions

For functional validation of these protein-encoding genes involved in carotenoid biosynthetic pathway in microalgae, a freshwater industrial strain *B. braunii* (Race B, NIES-836) procured from the Microbial Culture Collection at the National Institute for Environmental Studies (NIES Collection, Tsukuba, Japan) was cultured in BG-11 media at 24°C with 150 μE of light intensity and a photoperiod of 16:8 (L:D) at a constant shaking of 150 rpm (Singh et al., 2020). Growth was monitored by measuring optical density at 750 nm and by dry cell weight (DCW) analysis. Growth rates were obtained using the following equation:

(1) $$K=\frac{\frac{l\,n\,N\,2}{N\,1}}{t\,2-t\,1}$$

where N1 and N2 represent optical density at initial time (t1 = day 2) and final time (t2 = day 6) during the exponential phase, respectively. Doubling time was calculated depending on the specific growth rate.

(2) $$\text{Doubling}\,t\,i\,m\,e=\frac{\text{ln}\,2}{K}$$

Experiments demonstrating the channeling of carbon flux toward these energy rich molecules were screened as follows: BG-11 supplemented with bicarbonate (BG-11 + 0.08% NaHCO_3_), CO_2_ (BG-11 + 3% v/v CO_2_), and both (BG-11 + 0.08% NaHCO_3_ + 3% v/v CO_2_) against a control (BG-11) for 10 days for all further analyses and profiling. In the present study, CO_2_ was continuously bubbled in the culture medium, whereas bicarbonate was added as a single dose in the culture medium at the start of the experiment.

#### Quantification of Carotenoids Employing High-Performance Liquid Chromatography Analysis

Total pigments were estimated employing high-performance liquid chromatography (HPLC) analysis, and the extraction procedure was performed as described in Paliwal and Jutur (2021). Briefly, 10^6^ cells were centrifuged and resuspended in 1 ml of absolute methanol. For extraction of pigments, cell suspension was vortexed briefly with glass beads for 20 min. Supernatant was collected and used for HPLC-UV analysis carried out through Agilent Infinity series 1,260 HPLC system (Agilent Technologies, Santa Clara, CA, United States). The samples were run through a C30 Acclaim column (4.6 × 250 mm, 5 μm) maintained at 35°C with the binary solvent system as the mobile phase consisting methanol as primary solvent A and methyl *tert*-butyl ether (MTBE) as solvent B. The run conditions were as follows: 2–20% B for an initial 10 min, followed by 20% B (10–12 min), 20–80% B (12–30 min), 80% B (30–32 min), and 80–2% B (32–35 min) (Gleize et al., 2012). Pigments were detected at 437 nm and identified by comparing the retention time of the standards obtained from DHI, Hørsholm, Denmark.

#### Chlorophyll *a* Fluorescence Measurement

Chlorophyll fluorescence was estimated using Dual-PAM-100 fluorometer (Heinz Walz GmbH, Pfullingen, Germany). Samples were kept in the dark and incubated for a period of 30 min to ensure complete oxidation of all the reaction centers. The photosynthetic parameters were estimated as described previously (Kareya et al., 2020). For PSI: Y(I) = (Pm′–P)/Pm, Y(NA) = (Pm–Pm′)/Pm, Y(ND) = P/Pm as described by Klughammer and Schreiber (1994); Baker (2008), and Fang et al. (2020). The P700^+^ signals (P) could range from a minimum (P700 entirely reduced) to a maximum level (P700 fully oxidized), where P denotes P700^+^ signals, Pm is P700 fully oxidized, and Pm′ is P700 fully reduced.

#### Extraction and Quantification of Squalene

Squalene content was quantified as described by Kajikawa et al. (2015). Microalgal cells (10^9^ cells) were saponified in 2 ml of 10% KOH prepared in 50% methanol for 30 min by sonication. Squalene was extracted with the same volume of hexane, the solvent was evaporated, and the leftover dried residue was dissolved in 20 μl of chloroform. Derivatization was done by adding 20 μl of *N*,*O*-bis(trimethylsilyl)trifluoroacetamide (Sigma-Aldrich, St. Louis, MO, United States) to the samples and incubated at 80°C for 30 min. Chloroform was added to the derivatized samples to increase the reaction volume to 40 μl. A 2 μl aliquot of the solution was analyzed using gas chromatography–mass spectrometry (GC-MS) (Agilent Technologies, Santa Clara, CA, United States) equipped with an DB-5 MS capillary column (30 m × 0.25 mm × 0.25 mm). The carrier gas used in the experiment was helium with a flow rate of 1 ml min^–1^, and the initial oven temperature was 150°C, which was increased to 300°C (ramp rate 20°C min^–1^). The ionization voltage was 70 eV, and scan range was 40–500 Da. The squalene content was calculated from the ratio of the peak areas of the standard procured from Sigma-Aldrich, United States.

### Statistical Analysis

All the experiments were performed as biological triplicates and are represented as average ± SE. Statistical analyses such as ANOVA and *t*-test were performed using Microsoft excel for determination of significance.

## Results

### *In silico* Analysis of Methyl Erythritol Phosphate Pathway

The schematic representation of MEP pathway for the biosynthesis of squalene and carotenoids is illustrated in Figure 1. The first committed step of MEP pathway is catalyzation by DXS1, which results in the production of deoxy-xylulose phosphate. Isopentenyl pyrophosphate, the five-carbon isoprenoid precursor, condenses to form geranyl diphosphate, which has two fates: either it can be converted to phytoene through phytoene synthase (PSY), or it can be converted to squalene through two-step reaction catalyzed by farnesyl diphosphate farnesyl synthase (FPPS).

### Prediction of the Subcellular Localization for Protein-Encoding Genes

**FIGURE 2:**
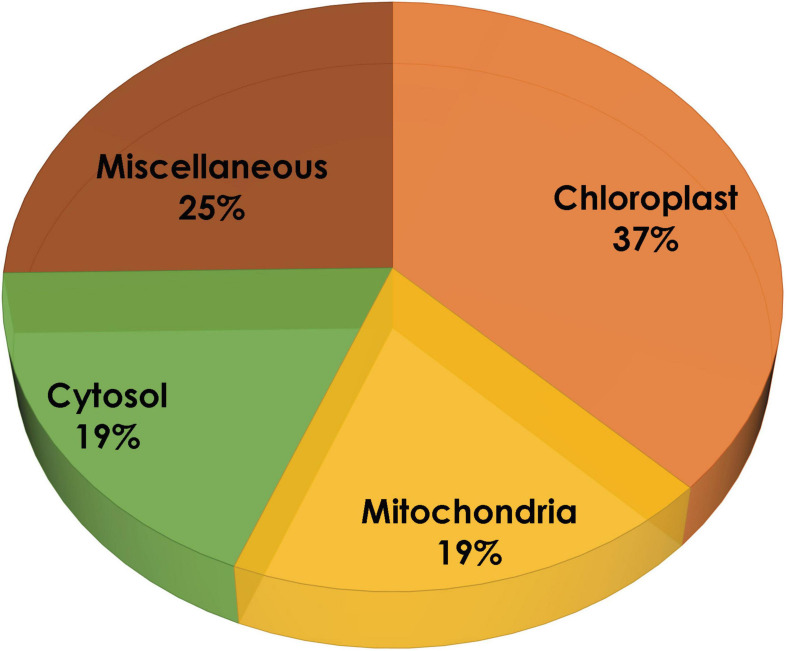
Subcellular localization of methyl erythritol phosphate (MEP) pathway genes using TargetP, WoLF PSORT, ngLOC, and Cello.

In the present study, the compartmentalization of all the proteins was predicted using four different tools: Cello, TargetP, WoLF PSORT, and ngLOC. These tools use different algorithms to predict subcellular localization for a particular protein, in order to obtain more reliable results based on the average data plotted. It is evident from Figure 2 that 59% of proteins are predominantly present in the chloroplast, 12% remain systematically organized in the cytoplasm, 13% of proteins are in mitochondria, and the remaining 16% are predicted to be localized in other compartments (like the nucleus, endoplasmic reticulum, and plasma membrane). As reported earlier, our study also predicts the localization of proteins involved in MEP pathway majorly within the plastids.

### Characterization of the Physico-Chemical Properties

**FIGURE 3:**
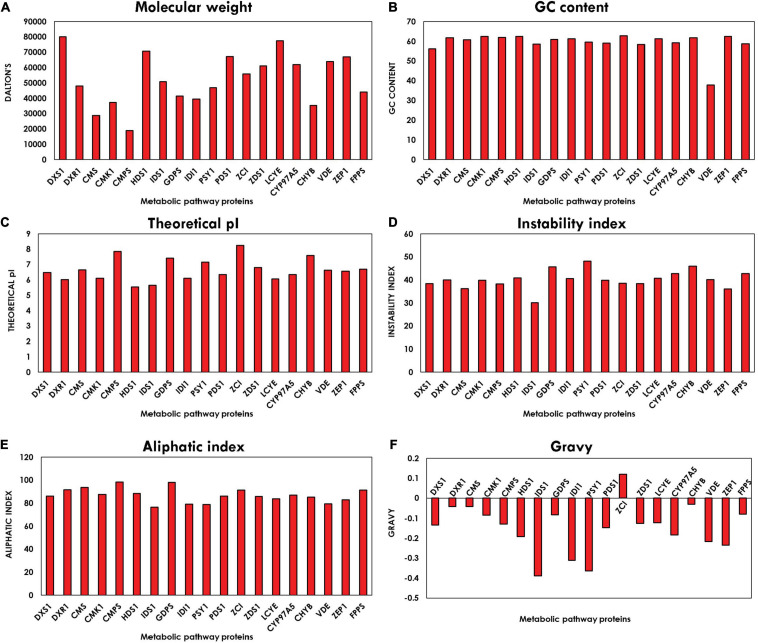
Bar plot depicting physico-chemical characteristics: **(A)** molecular weight **(B)**, guanine–cytosine (GC) content, **(C)** isoelectric point (pI), **(D)** instability index, **(E)** aliphatic index, and **(F)** grand average of hydropathy (GRAVY) index of methyl erythritol phosphate (MEP) pathway genes using ExPASy-ProtParam tool.

The physico-chemical properties characterized using Expasy’s ProtParam tool compute various parameters of proteins like instability index, molecular weight, pI, aliphatic index, and GRAVY index. The graph plotted from the average values of all parameters corresponding to all the proteins is shown in Figure 3. Molecular weight is observed between the range of 18,916.14 and 80,004.235 Da. The pI of the orthologous proteins lies in the range of 5–8, and majority of the proteins have their pI below 7, which indicates that they are acidic in nature. These values of the pI prove to be valuable and beneficial for developing a buffer system for purification of enzymes. Instability index defines the stability of proteins in their *in vitro* conditions. The existence of particular dipeptides occurring at significantly different frequencies between stable and unstable proteins is revealed by the instability index. Majority of the protein-encoding genes are stable, which are ideal prospects for genetic engineering involved in enhancement of biorenewables. The aliphatic index refers to the percentage of a protein’s volume filled by aliphatic side chains (alanine, leucine, isoleucine, and valine) and contributes to the globular proteins’ high thermal stability (Ikai, 1980). The average aliphatic index of all the proteins ranged from 76.49 to 98.47, with proteins with a high aliphatic index indicating structural stability over a wide temperature range. The GRAVY value for a protein is the sum of hydropathy value 10 of all the amino acids divided by the number of residues in the sequence. The GRAVY index can be used to measure the hydrophobicity of a protein. It is clearly evident from the plot in Figure 3 that all the proteins are hydrophilic, as their GRAVY index lies below zero. The GC content plot in Figure 3 shows the average GC content of all the genes ranging between 56 and 63%, which demonstrates the presence of a very high GC content.

### Identification of Motif and Domain Organization

**FIGURE 4:**
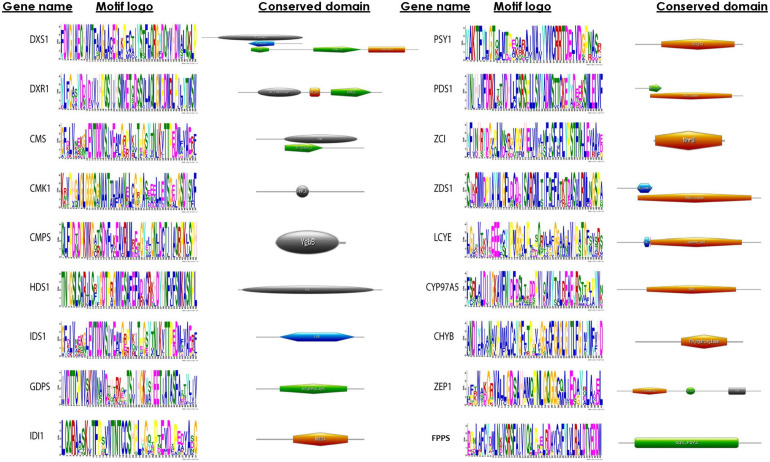
Conserved sequence logo plots representing motifs and domain architecture of methyl erythritol phosphate (MEP) pathway genes constructed using MEME suite and HMMER tool, respectively. In the sequence logo plot, the height of symbol represents the relative frequency of amino acid conserved at that position, whereas the color corresponds to nature of amino acid (such as DE, magenta; KR, red).

A motif is a pattern of sequence that is found conserved among a group of related protein or sequence. MEME algorithm is used widely for the discovery of DNA and protein sequence motifs. This computational tool predicts the conserved pattern amid the proteins, and result is depicted in a form of logo plot. In a sequence logo plot, the height of each stack indicates the relative occurrence of the corresponding amino acid, while the color indicates the nature of the amino acid. In addition to motif analyses, a detailed comparison of the domain architectures of the proteins was performed using HMMER. In this study, we were successful in identifying motifs of all the proteins involved in the carotenoid biosynthesis (Figure 4). Our analysis of domains revealed that phytoene desaturase (PDS1) and carotene desaturase (ZDS1) showed amino oxidase domain. Deoxy-D-xylulose-5-phosphate synthase (DXS), deoxy-D-xylulose 5-phosphate reductoisomerase (DXR), and 4-hydroxy-3-methyl-butenyl 1-diphosphate reductase displayed highly conserved motifs. HDS1 and IDS1 both were predicted to have GcpE domain, indicating that the proteins still retained the domain during the course of evolution. The domains in all these proteins show a pattern of high conservation among the microalgal species.

### Phylogenomic Analysis

**FIGURE 5:**
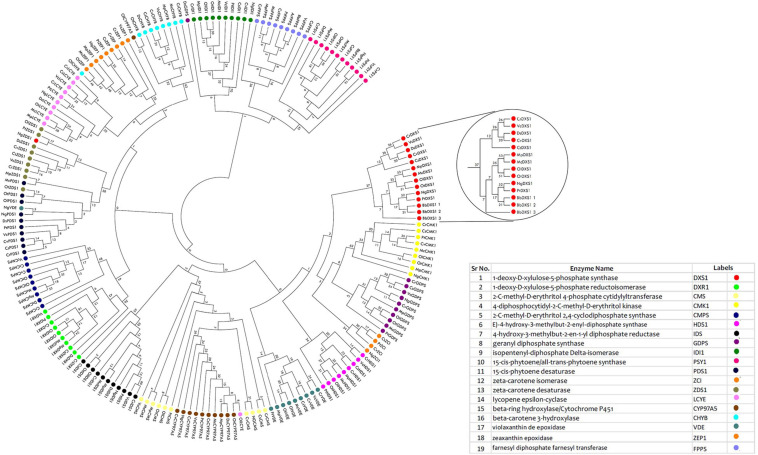
Phylogenomic tree constructed for methyl erythritol phosphate (MEP) pathway proteins employing neighbor-joining (N-J) method with evaluation of 1,000 rounds of bootstrapping test using MEGA 10.0. Each colored symbol denotes a specific protein, and the highlighted circle represents the rate-limiting enzyme deoxy xylulose phosphate synthase (DXS1) found to be conserved among microalgal species.

To understand the evolutionary relationship among the proteins involved in carotenoid and squalene synthesis among 13 species, a phylogenetic tree was constructed in the MEGA X software based on their protein sequences. The phylogenetic tree was constructed with 1,000 rounds of bootstrapping test in order to obtain exhaustive and detailed information. Homologs of proteins distributed among different species with similar functions were found to be clustered together in the tree as shown in Figure 5. Rate-limiting gene *DXS1* was found to be conserved among all the selected species, while three variants for this gene were retrieved in *B. braunii*. Proteins with the same domains were present in the neighboring clads as observed in phytoene desaturase and carotene desaturase, further proving the assumption that the domains and the sequence might be conserved in this protein during the course of evolution. The FPPS, a potential target gene for enhancement of squalene production, was found to be most conserved among chlorophytes as well as heterokont *Aurantiochytrium* sp. (which is a leading producer of squalene).

### Analysis of Subcellular Networking

**FIGURE 6:**
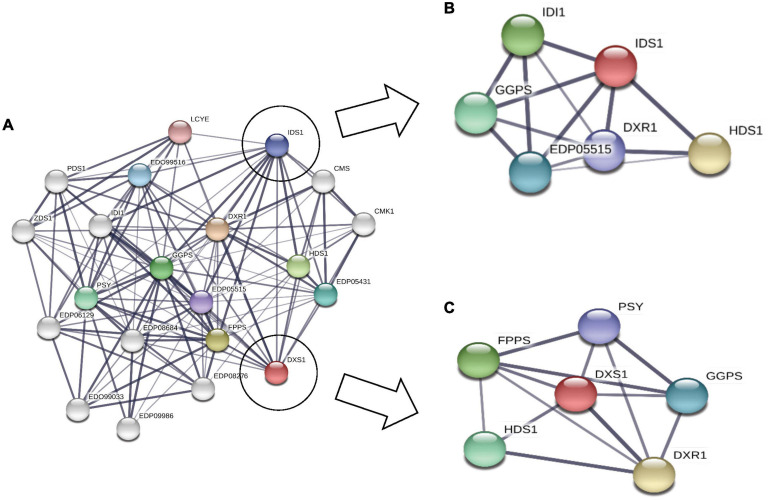
Subcellular network prediction using STRING database: **(A)** complete network where each node represents a particular protein of methyl erythritol phosphate (MEP) pathway protein; **(B)** subnetwork of IDS1 protein representing interaction with GGPS and DXR1; **(C)** subnetwork of DXS1 protein interacting with farnesyl diphosphate farnesyl synthase (FPPS) (squalene biosynthesis) and phytoene synthase (PSY) (carotenoid biosynthesis).

The regulatory network for MEP pathway proteins, which is predicted using STRING database (Figure 6), clearly shows the interaction between DXS1 and IDS1 proteins (gene involved in IPP synthesis pathway). Similarly, the rate-limiting enzymes for both carotenoid and squalene biosynthesis, i.e., PSY and FPPS, were found to be interacting, thus suggesting strong interaction and crosstalk among carotenoid and squalene synthesis pathway.

### Functional Characterization of Methyl Erythritol Phosphate Pathway in Green Alga *Botryococcus braunii*

The green microalga *B. braunii* was cultivated in the above-mentioned four conditions, i.e., in the presence of bicarbonate and CO_2_ supplementation. Table 1 represents the growth profile of *B. braunii* and shows that it is a slow-growing strain with a specific growth rate of 0.21 (day^–1^), doubling time of 3.37 days, and biomass productivity of 70 mg L^–1^ D^–1^. The growth rate has increased in the presence of NaHCO_3_, i.e., 0.23 (day^–1^), and a doubling time of 3.04 days and 77 mg L^–1^ D^–1^, but the change was not significant. In the presence of 3% CO_2_ alone, the biomass productivity increased 1.2-fold, i.e., 90 mg L^–1^ D^–1^ with the specific growth rate of 0.26 (day^–1^) and doubling time of 2.65 days. However, the addition of both bicarbonate and CO_2_ sparging has shown to be more promising than either of the factors alone and showed a 1.4-fold increase in the biomass productivity (100 mg L^–1^ D^–1^), although the specific growth rate and doubling time remains to be similar in the CO_2_ supplementation.

**TABLE 1:** Growth parameters of *Botryococcus braunii* subjected to carbon supplementation.

Conditions	Specific growth rate (μ) (day^–1^)	Doubling time (days)	Biomass productivity (mg L^–1^ D^–1^)
BG-11 (control)	0.21 ± 0.02	3.37 ± 0.08	70.99 ± 2.14
BG-11 (+NaHCO_3_)	0.23 ± 0.01	3.04 ± 0.03	76.86 ± 2.29
BG-11 (+3% CO_2_)	0.26 ± 0.06	2.65 ± 0.02	89.51 ± 2.84
BG-11 (+NaHCO_3_ + 3% CO_2_)	0.26 ± 0.04	2.70 ± 0.05	100.03 ± 2.44*

*Statistical significance by one-way ANOVA, p < 0.05.

### Dynamics of Chlorophyll *a* Fluorescence

We estimated the transient regulation of chlorophyll fluorescence using Dual-PAM, and different parameters for the photosynthetic efficiency of PSII and PSI were calculated and listed in Table 2. The maximum quantum efficiency of PSII photochemistry (F_v_/F_*m*_) of the cultures supplemented with CO_2_ and bicarbonate were found to be higher. The data predict the photosynthetic machinery in control conditions to be less responsive than cultures with additional carbon supplementation. The PSII operating efficiency, which can also be used as a proxy for the approximation of linear electron flux through the PSII, also seems to be enhanced in carbon-supplemented cultures. It is evident from Table 2 that the energy allocation is higher for NPQ in control conditions as compared with cultures with carbon supplementation. Furthermore, there is a distinct increase in the quantum yield of PSII photochemistry in cultures with additional carbon source. These results further indicate that the cells supplemented with carbon have an enhanced linear electron flow that supports the lowering of doubling time and enhanced growth rates for *B. braunii*. The quantum yield of PSI, denoted as Y(I), was distinctly more limited due to acceptor side limitation. Carbon supplementation increased the Y(I), while there was a decline in the control condition.

**TABLE 2:** Photosynthetic efficiency of *Botryococcus braunii* cultivated in different carbon supplementations.

Parameters	BG-11 (Control)	BG-11 (+NaHCO_3_)	BG-11 (+3% CO_2_)	BG-11 (+NaHCO_3_ + 3% CO_2_)
F_v_/F_*m*_	0.62 ± 0.02	0.74 ± 0.02	0.77 ± 0.01*	0.74 ± 0.02
Y(II)	0.15 ± 0.09	0.44 ± 0.003	0.42 ± 0.02	0.45 ± 0.01
Y(NPQ)	0.66 ± 0.09*	0.38 ± 0.02	0.31 ± 0.00	0.31 ± 0.00
Y(NO)	0.19 ± 0.01	0.18 ± 0.02	0.27 ± 0.02	0.24 ± 0.01
F_*q*_′/F_*m*_′	0.18 ± 0.01	0.44 ± 0.06	0.42 ± 0.02	0.45 ± 0.01
Y(I)	0.37 ± 0.01	0.49 ± 0.01	0.71 ± 0.01*	0.694 ± 0.01
Y(ND)	0.15 ± 0.01	0.11 ± 0.02	0.17 ± 0.01	0.22 ± 0.00*
Y(NA)	0.48 ± 0.01*	0.40 ± 0.02	0.12 ± 0.00	0.09 ± 0.01
ETR(I)	29.8 ± 0.80	39.4 ± 0.80	61.9 ± 1.40*	55.4 ± 1.60

Abbreviations for the labels included in the table are as follows: F_v_/F_m_, maximum photochemical efficiency of PSII; F_q_′/F_m_′, PSII operating efficiency; Y(II), quantum yield of photochemical quenching; Y(NPQ), quantum yield of non-photochemical quenching; Y(NO), energy dissipated as heat or fluorescence; Y(I), quantum yield of photochemical energy conversion; Y(ND), quantum yield of non-photochemical energy dissipation due to donor side limitation; Y(NA), quantum yield of non-photochemical energy dissipation due to acceptor side limitation; ETR(I), electron transport rate through PSI. * Statistical significance by one-way ANOVA, p < 0.05.

### Quantification of Squalene and Carotenoids

**FIGURE 7:**
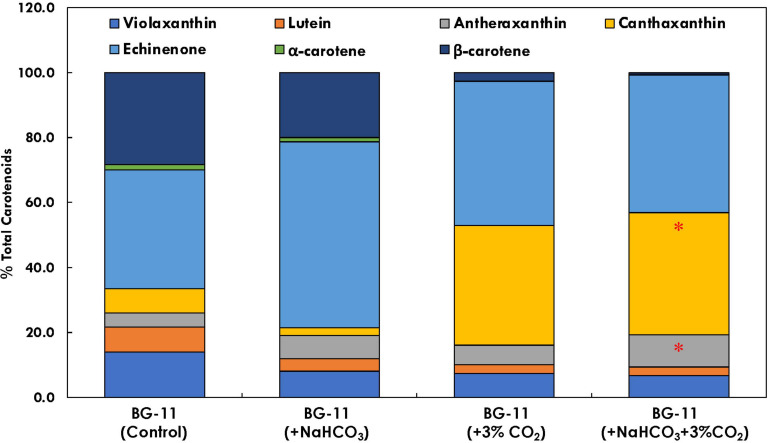
Bar plot depicting the distribution of individual carotenoids (% total carotenoid content) in *Botryococcus braunii* subjected to different carbon supplementations. * Statistical significance by one-way ANOVA, *p* < 0.05.

The carotenoid profile of *B. braunii* was found to be affected in the presence of both bicarbonate and CO_2_ supplementation (Figure 7). It has been found that carbon supplementation enhances chlorophyll content, which further enhances the photosynthetic efficiency and reduces the doubling time. In the presence of bicarbonate alone, echinenone was found to be 57.3% of total carotenoids, which was 1.5 times higher (*p* < 0.05) as compared with control. As compared with CO_2_ supplementation, there was significant difference in the carotenoid profile for cultures supplemented with both CO_2_ and bicarbonate. The antheraxanthin content has increased from 6.6% of total carotenoids in CO_2_ supplementation to 9.9% in the presence of both bicarbonate and CO_2_. Also, the β-carotene content was found to be the lowest in the presence of bicarbonate and CO_2_, which was significantly higher in the presence of bicarbonate alone (20% of total carotenoid content).

Table 3 represents the content of different HVRs obtained for *B. braunii* in the presence of NaHCO_3_ (0.8% w/v) and CO_2_ (3% v/v) on the 10th day. The squalene content significantly decreased in the carbon supplementation from 0.32% of DCW in control, 0.16% in NaHCO_3_, 0.06% in CO_2_, and 0.08% (*p* < 0.05) in the presence of both. However, the content of another HVR botryococcene was found to increase from 0.023% DCW in control to 0.055% DCW with bicarbonate and CO_2_ supplementation.

**TABLE 3:** The high value-added renewable profile of *Botryococcus braunii* on the 10^th^ day supplemented with different carbon sources.

HVRs (% DCW)	BG-11 (control)	BG-11 (+NaHCO_3_)	BG-11 (+3% CO_2_)	BG-11 (+NaHCO_3_ + 3% CO_2_)
Squalene	0.326 ± 0.02*	0.014 ± 0.06	0.006 ± 0.01	0.008 ± 0.05
Carotenoids	0.059 ± 0.01	0.036 ± 0.08	0.068 ± 0.03	0.180 ± 0.02*
Botryococcane	0.023 ± 0.05	0.021 ± 0.05	0.014 ± 0.03	0.055 ± 0.07

HVRs, high-value renewables; DCW, dry cell weight. *Statistical significance by one-way ANOVA, p < 0.05.

## Discussion

Recently, microalgae attracted considerable interest worldwide as a promising feedstock for biofuels and various high-value biorenewables. Microalgae are preferred over higher plants due to their high photosynthetic efficiency, greater ability to fix carbon dioxide (CO_2_) and convert CO_2_ into biomass, and shorter life cycle. The potential of several microalgae species in the renewable energy, biopharmaceutical, and nutraceutical sectors has been evaluated (Khan et al., 2018; Sun et al., 2018). Microalgae are an excellent source of HVRs such as polysaccharides, carotenoids, sterols, terpenoids, OMEGAs, and proteins, which are beneficial to human health (Chew et al., 2017; Shaikh et al., 2019). Simultaneous production of specific high-value compounds along with biofuels in a biorefinery concept could make the process economically feasible (Carriquiry et al., 2011; Campenni et al., 2013; Nobre et al., 2013). The major strategy applied for the enhancement of biofuel is stress biology aspects, which retard the biomass production. Another factor that can be applied for enhancing biomass along with certain other high-value products is carbon supplementation, which paved new way for increased production of HVRs (Markou and Nerantzis, 2013). Therefore, the biotechnological application in microalgae is limited under industrial conditions for the lack of comprehensive understanding of metabolic pathways and their regulation.

In the present study, phylogenomic analysis carried out for the MEP pathway is responsible for the production of carotenoids and squalene along with providing isoprenoid backbone to variety of other HVRs such as tocopherols (vitamins) and sterol. The MEP pathway genes of *C. reinhardtii* were retrieved and aligned with 13 other microalgal strains belonging to different classes of Chlorophyceae and Heterokonts. The nucleotide sequence for the hits obtained is used for understanding the sequence similarity and evolutionary gene–function relationship among various microalgal species.

IPP is a common precursor for the production of majority of HVRs. These compounds, apart from having commercial relevance, also impart certain benefits to organisms themselves, and hence, a detailed investigation of isoprenoid pathway will be crucial for further tuning the HVR production. In plants, IPP is synthesized *via* two different pathways: firstly, the cytoplasmic mevalonate (MVA) pathway that initiates with the condensation of three units of acetyl-CoA to 3-hydroxy-3-methylglutaryl-CoA (HMG-CoA) and subsequently reduction to MVA, followed by successive phosphorylation of MVA, and a decarboxylation/elimination step leading to IPP (Lange et al., 2000). Secondly, IPP, which is derived from 2-*C*-methyl-D-erythritol 4-phosphate (MEP) pathway, occurs in chloroplast. The first step of this pathway is the condensation of pyruvate with the aldehyde group of D-G3P leading to the production of 1-deoxy-D-xylulose 5-phosphate (DXP), which is catalyzed by a thiamine-dependent synthase, i.e., 1-deoxy-D-xylulose 5-phosphate synthase (DXS). An intramolecular rearrangement and reduction of DXP by the enzyme DXP reducto-isomerase (DXR) yields 2-*C*-methyl-D-erythritol 4-phosphate (MEP) in the second step. This follows the conversion of MEP into 2-*C*-methyl-D-erythritol 2,4-cyclodiphosphate (ME-2,4cPP) in three enzymatic steps, and the subsequent reduction produces 1-hydroxy-2-methyl-2-butenyl 4-diphosphate (HMBPP), catalyzed by HMBPP synthase (HDS). This HMBPP is finally converted into a mixture of IPP and dimethylallyl diphosphate (DMAPP) by the enzyme HMBPP reductase (Carretero-Paulet et al., 2010), and the interconversion of IPP and DMAPP is controlled by IPP isomerase.

Majority of the microalgae were reported to have only MEP pathway for the production of IPP molecules that were further confirmed by labeling experiments (Rosa Putra et al., 1998). However, there are reports that rhodophyte possesses both the pathways, whereas chlorophyte has only MEP pathway (Lichtenthaler et al., 1997). Hence, there is load on plastid for generation of abundant pool of IPP, which is efficiently transported to the cytoplasm for sterols and other high-value products.

The two pathways for carotenoid and squalene bifurcate from GPP, one of which is a multistep pathway for the synthesis of the former, while the latter one is a two-step pathway. Figure 1 shows a complete picture of the carotenoid and squalene biosynthetic pathway along with the proteins present (DellaPenna and Pogson, 2006). It was revealed (Supplementary Table 1) that algal species maintain the basic genomic repertoire required for the production of isoprenoids. Furthermore, subcellular localization of proteins was performed, which gives an idea of its spatial organization and improves our knowledge of cellular metabolism. It also helps us to determine subcellular network topology. Our analysis revealed that 59% of the total proteins were localized in chloroplast, which as well supported by previous literature (Gong and Bassi, 2016). The IPP pathway enzymes are nucleus encoded and transported into plastids post-translationally, as evidenced by the presence of characteristic N-terminal transit peptides (Lohr et al., 2012).

The physico-chemical properties of proteins provide insight into the stability and functionality of proteins, further increasing the information regarding protein, helping in maintaining the structure, function, and the stability of the proteins in molecular work. For example, proteins having an instability index of less than 40 are considered stable, whereas those with a value of more than 40 are considered unstable (Misra et al., 2012). Our analysis revealed that the majority of the proteins are highly stable with high GC content, which makes it difficult for genetic manipulation. Furthermore, the motif and domain architecture revealed the evolutionary conservation of the proteins among the microalgae. The gene having similar domain were found to cluster together in the phylogenomic tree (Figure 5), which was constructed to understand the evolutionary relationship. Some of the interesting observations of this study were occurrences of three variants of DXS1; the rate-limiting gene of MEP pathway in *B. braunii* and only FPPS gene was found to align for *Aurantiochytrium* sp.

*Botryococcus* are green photosynthetic microalgae with the ability to constitutively synthesize, accumulate, and secrete substantial amounts of hydrocarbons such as alkadienes (A-race) or tri-terpenoids (B-race) and have the ability to synthesize odd-numbered hydrocarbons (C-23–C-33) (Jin et al., 2016). The green microalga can produce hydrocarbons in the range of 2–86% (% DCW) depending among strains/races and changes in cultural and physiological conditions (Rao et al., 2012). Despite the high hydrocarbon content, this microalga cannot be utilized on a larger-scale due to its slow growth. Therefore, in order to improve biomass production, the alga requires supplementation of carbon source (CO_2_, glucose sodium acetate, etc.) (Yoshimura et al., 2013; Barajas-Solano et al., 2016). In the present study, we have used two carbon sources, i.e., 0.08% (w/w) NaHCO_3_ and 3% CO_2_ (v/v), and a combination of both to enhance their biomass productivity.

Our preliminary analysis revealed that the green microalga is a slow-growing strain that has a doubling time of 3.2 days (Table 1). However, the supplementation of CO_2_ reduces doubling time significantly, i.e., 2.6 days. Previous studies have suggested that adding carbon to microalgae culture is a major determinant, as excessive concentrations can inhibit growth or accumulation of particular metabolites, while low quantities might restrict it (Tapie and Bernard, 1988; Olaizola et al., 1991). As a result, carbon source optimization is required, which varies by species. Concentrations must not only be lower than a particular value that meets the algae’s carbon needs but also not exceed this value in order to avoid a significant loss that can ultimately lead to waste and significantly rise of production cost (Cheng et al., 2006). Yoshimura et al. (Yoshimura et al., 2013) screened different concentrations of CO_2_ from 0.02 to 5% for cultivation of *B. braunii* Showa and found that the specific growth rate remained similar for the aforementioned CO_2_ concentrations; however, growth has declined above 5%, which may be due to the drop in pH. This further supports the enhanced biomass productivity of 100 mg L^–1^ D^–1^ with a combination of both bicarbonate and CO_2_ supplementation.

It has been reported that biomass concentration increases in cultures supplemented with additional source of carbon (Sforza et al., 2012; Adamczyk et al., 2016). Moreover, the chlorophyll content along with the photosynthetic efficiencies appears to be enhanced upon CO_2_ supplementation in *M. gaditana*, further lowering the doubling time (Kareya et al., 2020). We demonstrate that the additional supplementation of carbon in *B. braunii* significantly reduces the doubling time, which is accompanied by higher chlorophyll content F_v_/F_*m*_ ratio. The maximum quantum efficiency of PSII remains similar in cultures supplemented with carbon source and is ∼1.2-fold higher than control conditions. Similarly, the PSII operating efficiencies of cells supplemented with carbon are clearly higher (∼4.0-fold) than those of control. The increase in quantum efficiency of PSII along with PSII operating efficiency, which provides an estimate of the quantum yield of linear electron flux through PSII, is also reported to be due to the synergistic effect of certain pigments on the photosystems (Kareya et al., 2020). The low photochemical efficiencies in control cultures further influence the lack of effective electron flux through the photosystems, and this manifestation is also noticeable in the growth of all the cultures.

The quantum yield of non-photochemical quenching Y(NPQ) is prominently higher in the control condition compared with cells supplemented with carbon source. Conversely, the quantum yield of PSII photochemistry Y(II) is higher in cultures supplemented with carbon than in the control condition. As a result, we demonstrate that the carbon flux helps the cells to undergo regulated changes efficiently as compared with the control condition; moreover, due to the lack of carbon, control cells might undergo photoinhibition, suggesting that a greater number of PSII reaction centers are closed in the control condition (Fang et al., 2020; Lu et al., 2020). Carbon supplementation further helped in the increase in the Y(I), suggesting that the photosynthetic efficiency was stable and balanced as compared with that in the control condition. Furthermore, there was an increase in Y(NA) in the control condition, suggesting the limitation due to the acceptor side in the photosystems and high Y(ND) in carbon-supplemented cells displaying the necessity of carbon to inhibit the over-reduction of PSI electron carriers (Sun et al., 2020).

The xanthophyll and carotene compositions are crucial for photoprotection and PSI stability, as it helps in reactive oxygen species (ROS) scavenging and quenching of Chl^∗^ (Grung et al., 1994). The xanthophyll violaxanthin is rapidly de-epoxidized to intermediate antheraxanthin and zeaxanthin in order to convert conversion of PSII to a state of high thermal energy dissipation and low Chl fluorescence emission, hence lowering the photoinhibition (Havaux and Niyogi, 1999). In the presence of carbon supplementation, antheraxanthin accumulation was found (Figure 7), which relates with the lower NPQ and higher biomass accumulation. Additionally, the primary carotenoids α- and β-carotenes were found to be lower in the presence of additional carbon; however, canthaxanthin was higher. This may be attributed to the upregulation of lycopene epsilon cyclase in the presence of carbon supplementation.

Two of the HVRs, carotenoid and squalene, were found to have an entirely opposite effect in the presence of carbon supplementation. The carotenoid content increased in the presence of carbon source: maximum for the 3% CO_2_ and NaHCO_3_, i.e., 0.18% of DCW (Table 3) with *p* < 0.05, whereas the squalene content has declined threefold from 0.32% in control to 0.08% in CO_2_ supplementation. Carotenoid content can be enhanced by employing various media engineering approach such as nutrient starvation, salinity, high light irradiance, and carbon supplementation (Paliwal et al., 2017). Also, there are certain regulatory proteins such as PSY and phytoene desaturase, which were overexpressed for enhanced carotenoid production in various plants and microalgal species. Wang et al. (2021) identified three variants of PSY in tobacco plant, i.e., PSY1, PSY2, and PSY3, and found that silencing of PSY1 and PSY2 remarkedly decreased chlorophyll and carotenoid content in leaves. In *Arabidopsis thaliana*, an ORANGE (OR) protein that regulates chromoplast differentiation was found to interact with PSY, and overexpression of OR significantly enhanced the enzymatically active PSY, which leads to enhanced carotenoid production (Zhou et al., 2015).

## Conclusion

**FIGURE 8:**
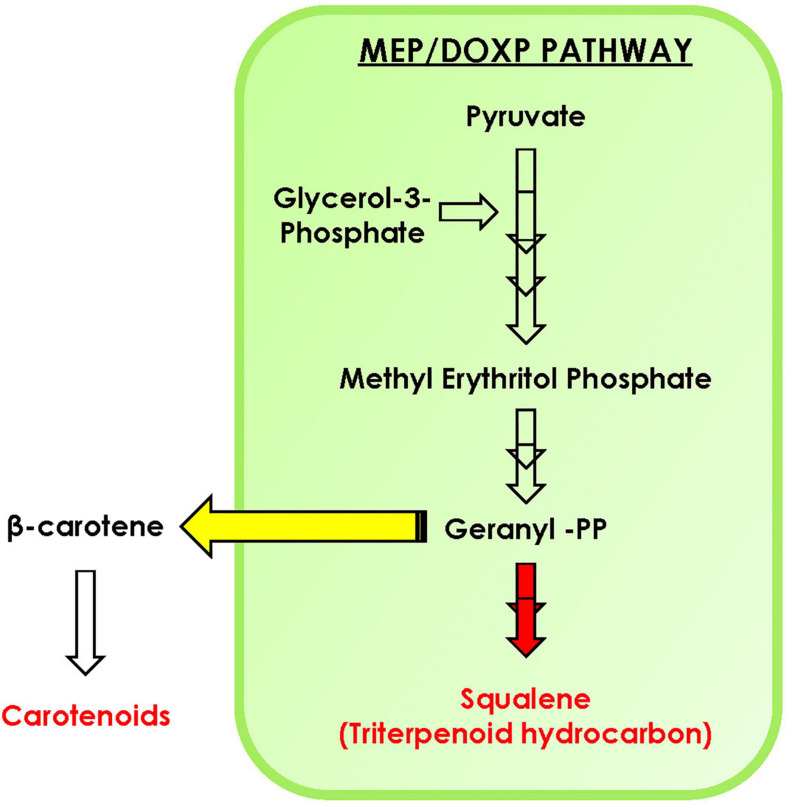
Schematic representation of carotenoid and squalene biosynthesis in *Botryococcus braunii*. Yellow arrow indicates the diversion toward carotenoids, while red indicates downregulation of squalene production upon carbon supplementation.

In conclusion, with the help of phylogenomic analysis, we demonstrate that isoprenoid pathway in the microalgal lineage is highly conserved and is highly regulated with complex crosstalk within the pathway. Furthermore, upon carbon supplementation in selected microalga *B. braunii*, doubling time declines significantly along with diverting the carbon flux toward accumulation of carotenoids, specifically lutein. The STRING analysis predicts the interaction between various proteins of MEP pathway such as DXS1, IDS1, GGPS, PSY, and FPPS. Overall, we hypothesize that the enhancement in carotenoid biosynthesis might be attributed toward the upregulation of PSY, whereas the FPPS was downregulated in the presence of external carbon source (Figure 8). Thus, engineering of such regulatory proteins may further enhance the carotenoid content of *B. braunii*. Our present study highlights for the first time the crosstalk between carotenoid and squalene biosynthesis pathway in the presence of carbon supplementation, a new perspective of media engineering a cost-effective approach to enhance production of biorenewables without compromising growth.

## Data Availability Statement

The original contributions presented in the study are included in the article/Supplementary Material, further inquiries can be directed to the corresponding author/s.

## Author Contributions

IM, AN, and PJ designed the experiments. IM, MR, and MK executed the experiments. AN and PJ supervised the project. IM, MR, MK, and AN wrote the manuscript with all the input from the authors. All authors contributed to the article and approved the submitted version.

## Conflict of Interest

The authors declare that the research was conducted in the absence of any commercial or financial relationships that could be construed as a potential conflict of interest.

## Publisher’s Note

All claims expressed in this article are solely those of the authors and do not necessarily represent those of their affiliated organizations, or those of the publisher, the editors and the reviewers. Any product that may be evaluated in this article, or claim that may be made by its manufacturer, is not guaranteed or endorsed by the publisher.

## Footnotes

1
https://www.genome.jp/kegg/KEGG

2
https://blast.ncbi.nlm.nih.gov/Blast.cgi

3
https://meme-suite.org/meme/tools/meme

4
http://hmmer.org

5
https://string-db.org/
